# Can Thrombosed Abdominal Aortic Dissecting Aneurysm Cause Mesenteric Artery Thrombosis and Ischemic Colitis?—A Case Report and a Review of Literature

**DOI:** 10.3390/jcm14093092

**Published:** 2025-04-29

**Authors:** Laurențiu Augustus Barbu, Nicolae-Dragoș Mărgăritescu, Liliana Cercelaru, Daniel-Cosmin Caragea, Ionică-Daniel Vîlcea, Valeriu Șurlin, Stelian-Ștefaniță Mogoantă, Gabriel Florin Răzvan Mogoș, Liviu Vasile, Tiberiu Ștefăniță Țenea Cojan

**Affiliations:** 1 Department of Surgery, Railway Clinical Hospital Craiova, University of Medicine and Pharmacy of Craiova, 2 Petru Rareş Street, 200349 Craiova, Romania; laurentiu.barbu@umfcv.ro (L.A.B.); gabriel.mogos@umfcv.ro (G.F.R.M.); tiberiu.tenea@umfcv.ro (T.Ș.Ț.C.); 2 Department of Surgery, Emergency County Hospital, University of Medicine and Pharmacy of Craiova, 2 Petru Rareş Street, 200349 Craiova, Romania; ionica.valcea@umfcv.ro (I.-D.V.); vsurlin@gmail.com (V.Ș.); ssmogo@yahoo.com (S.-Ș.M.); vliviu777@yahoo.com (L.V.); 3 Department of Pathology, University of Medicine and Pharmacy of Craiova, 200349 Craiova, Romania; liliana.cercelaru@umfcv.ro; 4 Departament of Internal Medicine, University of Medicine and Pharmacy of Craiova, 200349 Craiova, Romania; daniel.caragea@umfcv.ro

**Keywords:** ischemic colitis, abdominal aortic aneurysms, inferior mesenteric artery thrombosis

## Abstract

**Background/Objectives:** Ischemic colitis, typically caused by thrombosis or reduced blood flow in the inferior mesenteric artery, is the most common ischemic lesion at the colorectal level. This case contributes to existing knowledge by highlighting the rare co-occurrence of a thrombosed aortic aneurysm and ischemic colitis, pointing to a direct vascular etiology rather than a multifactorial or idiopathic cause. **Methods:** A thorough electronic search was conducted on PubMed to identify risk factors and etiological determinants of ischemic colitis. **Results:** We present the case of a 70-year-old male with diffuse abdominal pain and multiple cardiac comorbidities. A CT scan revealed aeroenteritis, aerocolia, fusiform aneurysmal dilation of the abdominal aorta (18 cm long, 7.3 cm in diameter, from below the renal arteries to the bifurcation), parietal thrombosis, a circulating lumen of 2.7 cm, and inferior mesenteric artery thrombosis. Intraoperatively, necrosis was found in the upper rectum, sigmoid colon, descending colon, and the middle third of the left transverse colon, with clear demarcation between healthy and necrotic tissue. A subtotal proctocolectomy with transverse colostomy was performed. **Conclusions**: This case highlights ischemic colitis as a vascular disorder, urging broader differential diagnosis when common causes are unclear. Timely imaging, a multidisciplinary approach, and attention to vascular risks are key to identifying rare causes like aneurysmal thrombosis. While thrombosed abdominal aortic aneurysms can cause mesenteric ischemia, their link to ischemic and ulcerative colitis is unique, emphasizing the importance of accurate risk assessment in treatment planning.

## 1. Introduction

Acute mesenteric ischemia is a severe and potentially deadly condition. It is caused by arterial obstruction from thrombosis (arteritis or atherosclerosis), trauma, rupture of a mesenteric aneurysm, embolism from the right atrium or abdominal aorta, and mesenteric venous thrombosis [1,2]. Low blood pressure from shock or heart failure can decrease intestinal blood flow, leading to mesenteric ischemia [1,3].

Mesenteric ischemia is a sudden reduction in intestinal blood flow, caused by either occlusive or non-occlusive disorders of the mesenteric vessels. A mesenteric aneurysm is a rare but serious dilation of an artery supplying the intestines, most often affecting the superior or inferior mesenteric artery.

Ischemic colitis, first described by Boley et al., results from thrombosis or reduced blood flow in the inferior mesenteric artery. The most common ischemic lesion occurs at the colorectal level [4,5], presenting as either obstructive or nonobstructive, with an incidence of 1 in 1000 hospitalizations. Diagnosis can sometimes be challenging due to transient forms that respond to anticoagulant therapy but carry a high risk of morbidity and mortality in ischemic gangrenous colitis [6]. Young individuals are rarely affected, with over 90% of colonic ischemia cases occurring in the elderly [4]. Ulcerative colitis is an autoimmune disorder caused by immune system overactivity, resulting in ulceration and inflammation in the colorectal region.

European studies have noted a rise in ulcerative colitis incidence among both young individuals and those over 65 [6,7,8,9,10]. Post-therapeutic outcomes in elderly ulcerative colitis patients are poor due to comorbidities, frailty, higher infection risk, and the potential for neoplasm development during biological therapies [6,11]. Ischemic colitis is an increasingly common and atypical condition, presenting with diverse clinical manifestations at diagnosis [12].

Abdominal aortic aneurysms are aortic dilations, typically asymptomatic, and most commonly found in the infrarenal segment [13,14]. Abdominal aortic aneurysms are present in elderly patients, with high morbidity and mortality, especially in the case of aneurysm rupture. Intraluminal thrombus is found in nearly all abdominal aortic aneurysms and can impact aneurysm wall repair. It leads to thrombosis, though the exact mechanisms remain unclear [13].

Inflammatory and hemodynamic factors, along with thrombosis, damage the tunica media of the aortic wall, causing irreversible pathological remodeling. This results in wall weakening, dilation, and ultimately rupture [13].

The wall of abdominal aortic aneurysms shows changes similar to atherosclerosis, including matrix damage and nonocclusive thrombus formation, along with some characteristic features of arterial thrombosis [13,15]. Although thrombus embolization is rare, it significantly increases morbidity and mortality when it occurs [13,16]. The atherothrombotic process in abdominal aortic aneurysms does not resolve or remodel arterial lesions, but can ultimately lead to rupture [13].

## 2. Case Report

A 70-year-old male was admitted with diffuse abdominal pain lasting 3 days. His medical history includes congestive heart failure (class II), hypertension, ischemic heart disease, right bundle branch block, and mitral valve insufficiency, with no previous abdominal surgeries. He was treated with angiotensin-converting enzyme inhibitors, beta blockers, aldosterone antagonists, angiotensin–neprilysin receptor inhibitors, and diuretics. His cardiac condition had been diagnosed years earlier, and he had regular follow-ups with a cardiologist, receiving medication as prescribed.

The abdominal examination revealed diffuse tenderness on palpation, slight abdominal swelling, absent bowel sounds, and signs of peritoneal irritation. Physical examination showed the patient’s general condition to be normotensive, tachycardic, and normoxemic. Laboratory tests indicated infection, with elevated leukocytes and neutrophils, suggesting the pathology had begun prior to the patient’s hospital admission (Table 1).

**Figure 1:**
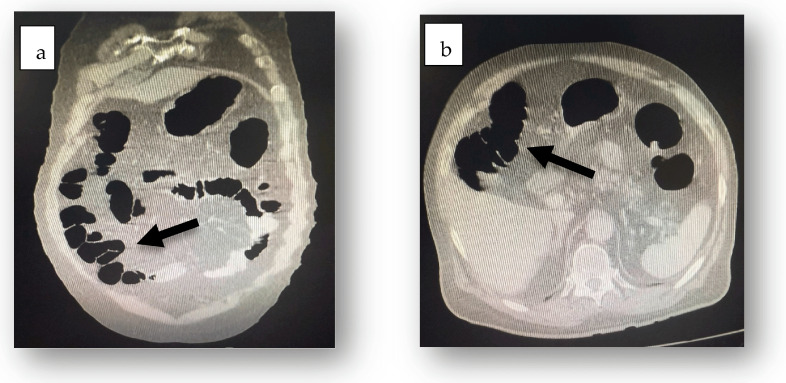
CT scan revealed intraluminal air in both the small bowel and colon (black arrow), consistent with aeroenteria (**a**) and aerocolia (**b**), respectively.

**Figure 2:**
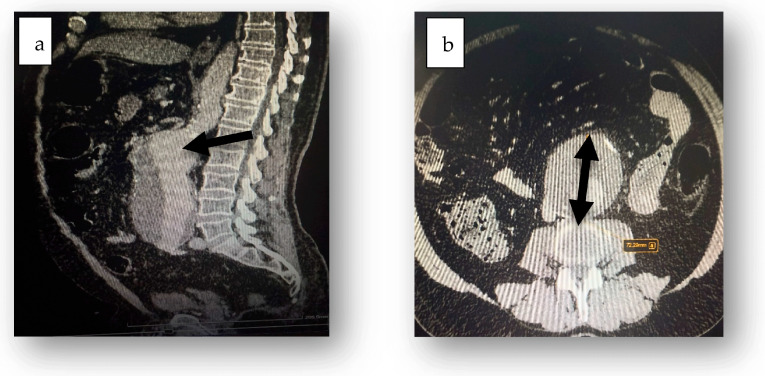
Fusiform aneurysmal dilation of the abdominal aorta (black arrow), extending 18 cm from below the renal arteries to the bifurcation (**a**), with a maximum diameter of 7.3 cm (**b**).

**Figure 3:**
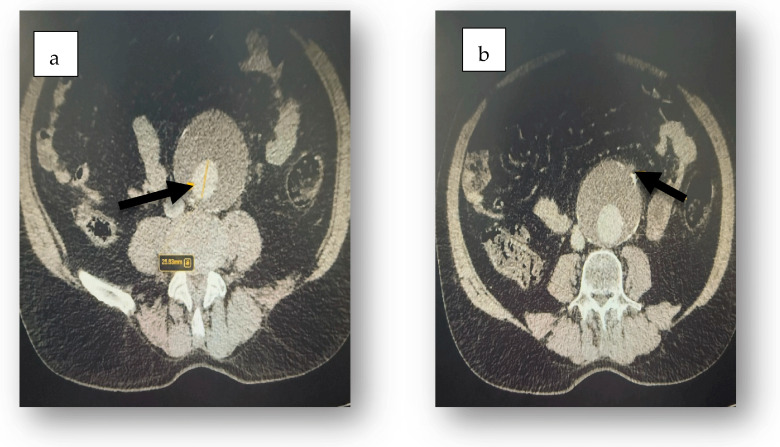
CT scan shows parietal thrombosis (black arrow) with a 2.7 cm circulating lumen (**a**) and thrombosis of the inferior mesenteric artery (black arrow) causing ischemic colitis (**b**).

Tests for *Clostridioides difficile* toxins (Toxin A and B) and glutamate dehydrogenase in fecal samples were negative. Abdominal ultrasound revealed accentuated abdominal meteorism without detectable peritoneal fluid. CT scanning revealed aeroenteria, aerocolia (Figure 1), a fusiform abdominal aortic aneurysm extending 18 cm with a maximum diameter of 7.3 cm, parietal thrombosis with a 2.7 cm circulating lumen (Figure 2), absence of contrast extravasation, and thrombosis of the inferior mesenteric artery (Figure 3).

The patient’s condition worsened, and he was transferred to the intensive care unit. Laboratory tests, including arterial blood gas analysis, revealed total respiratory compensated metabolic acidosis 6 h after admission. At 10 h post-admission, severe pure metabolic acidosis was observed (Table 2).

The pre-anesthetic examination indicated a poor health status, with the patient being cooperative and conscious. Blood pressure was 100 mmHg (under Gelofusine), AV was 50 mL, diuresis was 50 mL, and the ASA (American Society of Anesthesiologists) physical status was classified as 5E.

**Figure 4:**
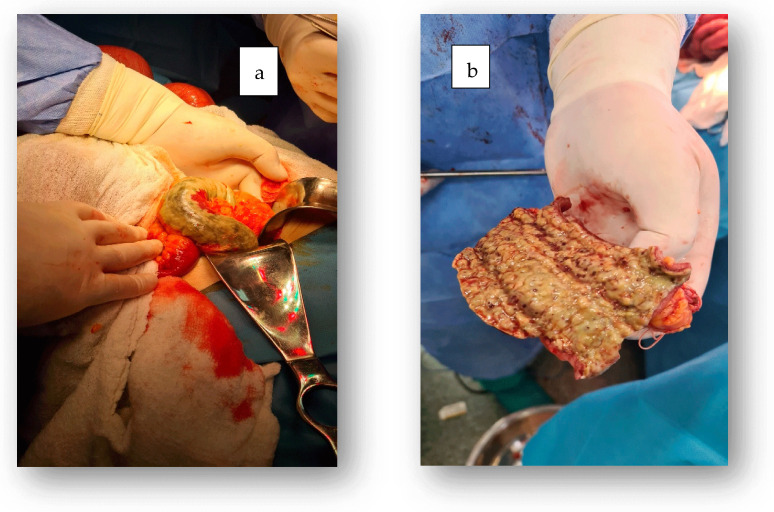
Intraoperative findings revealed serosal necrosis in some areas (**a**), and sectioning of the resected tissue showed colonic mucosal necrosis extending deep into the wall (**b**).

The patient underwent a median laparotomy. Intraoperatively, necrotic tissue was found in the upper rectum, sigmoid, descending colon, and the middle third of the left transverse colon, with a clear demarcation between healthy tissue and the necrotic area. A subtotal proctocolectomy with transverse colostomy was performed (Figure 4).

The patient’s condition deteriorated, with laboratory tests showing signs of sepsis (Table 2). He was intubated orotracheally and placed on continuous mechanical ventilation. Blood pressure was 60/40 mmHg, supported by noradrenaline. The patient experienced an unresuscitable cardiac arrest 12 h postoperatively.

Microscopic analysis of the colon was performed. Samples were fixed in 10% neutral-buffered formalin for 24 h, then embedded in paraffin. Hematoxylin and eosin staining was applied, and sections were examined under a Panthera L light microscope (Motic Europe, S.L.U, Barcelona, Spain) at various magnifications.

**Figure 5:**
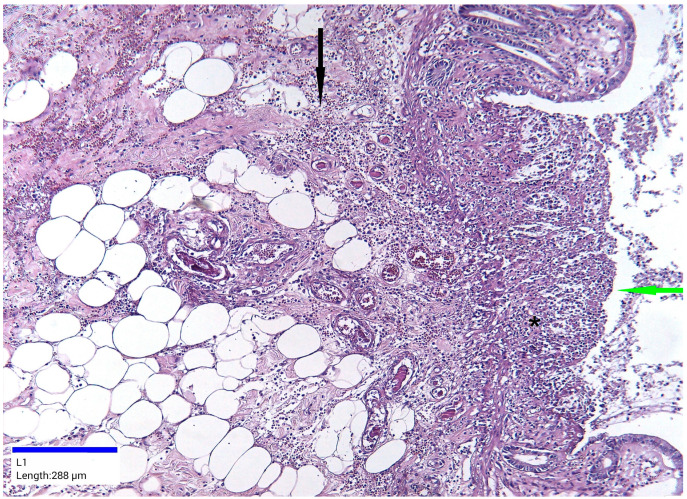
Ischemic colitis pattern, hematoxylin and eosin staining, ×10. Colonic mucosa and submucosa show areas of ulceration (green arrow), absence of crypts (black asterisk), foci of hemorrhage, and chronic inflammation (black arrow).

**Figure 6:**
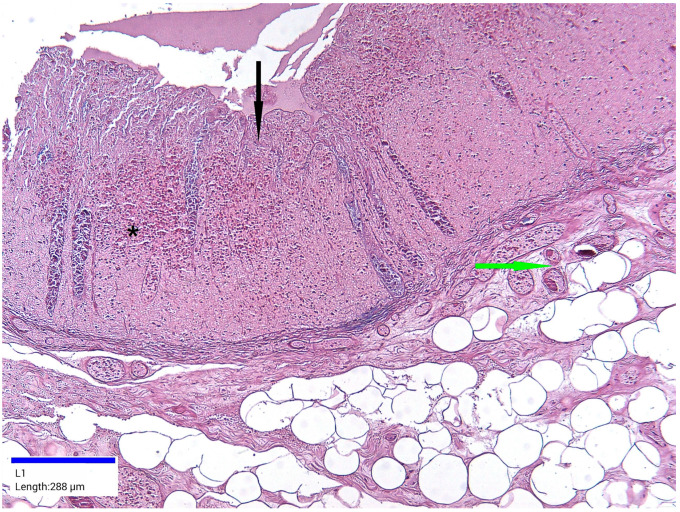
Ischemic colitis pattern, hematoxylin and eosin staining, ×10. Mucosal necrosis (black arrow) accompanied by hemorrhagic foci (black asterisk) and vascular congestion (green arrow).

Microscopic examination revealed various histopathological features in the colon. The pathological process affected both the mucosa and submucosa. Ulcerations were present in the mucosa, accompanied by architectural distortions, a reduction in the size and number of crypts, or their complete absence. Edema, foci of hemorrhage, and pink hyaline material were observed in the lamina propria. Vascular congestion, focal microthrombi, and chronic inflammation were noted in the mucosa, extending into the submucosa. In other sections, necrosis was observed extending into the submucosa (Figure 5 and Figure 6).

## 3. Discussion

A cardiac comorbid patient presented with 3 days of diffuse abdominal pain, with examination revealing tenderness and signs of peritoneal irritation. Labs revealed leukocytosis, thrombocytopenia, elevated inflammatory markers, and increased urea/creatinine. CT scanning showed a fusiform aneurysm with thrombus and mesenteric artery thrombosis, suggesting ischemic colitis. His condition worsened, and he was transferred to the intensive care unit. Emergency surgery revealed necrosis in the rectum, sigmoid, descending colon, and middle transverse colon, requiring subtotal proctocolectomy with transverse colostomy.

Microscopic analysis showed necrosis and ulceration, suggesting both ischemic and ulcerative colitis. The patient had no prior symptoms or diagnosis of ulcerative colitis, likely indicating an asymptomatic, slowly developing form.

Ischemic colitis is a surgical emergency with an incidence of 14% to 66% [2,3,4,5]. It can range from asymptomatic, medically reversible forms to severe gangrene, obstruction, and perforation requiring emergency surgery. Postoperative outcomes are worsened by pre-existing comorbidities, with morbidity and mortality rates ranging from 5% to over 80%, depending on the timing of diagnosis and surgery, as reported in the literature [2,5,6,7,8].

Tseng et al. found that patients undergoing emergency colectomy for ischemic colitis had a 30-day postoperative mortality rate of 25.3% [17]. The study also highlighted a high frequency of complications, including prolonged intubation (35.2%), pneumonia (13.5%), septic shock (26.3%), and acute renal failure (6%) [17].

Common predictive risk factors for mortality include male gender, advanced age, history of coronary and peripheral vascular disease, atrial fibrillation, dialysis dependence, and prior cardiovascular surgery [17,18]. Male gender is not a definitive predictor of mortality, though some studies suggest otherwise [17,18,19,20].

The absence of rectal bleeding on admission as a risk factor for delayed surgery and death is supported by studies from Hughier et al., Paterno et al., Moszkowicz et al., and Añón et al. [18,19,21,22].

Trauma, embolisms, and thrombi in the inferior mesenteric artery are common in colonic ischemia. Risk factors like peripheral hypoperfusion from decompensated heart failure, transient hypotension, septic shock, and hypovolemia contribute to the development of ischemic colitis [4,23]. Tumors, postoperative adhesions, volvulus, hernias, and diverticula are rarely causes of ischemic colitis [4,23]. Ischemic colitis lesions are also uncommon after colonoscopy and colon surgery [4,23]. Certain drugs, including antibiotics, hormonal treatments, chemotherapy, diuretics, serotonin agents, vasopressors, narcotics, statins, psychotropics, and NSAIDs, are associated with ischemic colitis [4,24].

Ischemic colitis is a complication of coronary artery bypass and abdominal aortic aneurysm repair, occurring in 2–3% of cases [4,23]. Genetic factors, including deficiencies in protein C, S, and Z, antithrombin, factor V Leiden mutation, prothrombin mutation (20210G/A), and antiphospholipid antibodies, have been linked to ischemic colitis [25,26,27,28,29,30,31,32].

In healthy young patients without a clear cause of ischemia, ischemic colitis is linked to cocaine and methamphetamine use, long-distance running, vasculitis, and estrogen use [4]. Theodoropoulou et al. identified the 506 Q allele of the factor V (FV) Leiden mutation and the mutant 4G allele of plasminogen activator inhibitor (PAI) polymorphism in ischemic colitis patients [33]. While the role of a hypercoagulable state in ischemic colitis is not fully understood, certain genetic defects are linked to its occurrence [34].

Koutroubakis et al. studied thrombotic risk factors in ischemic colitis, finding frequent associations with antiphospholipid antibodies and factor V Leiden mutation [32]. Midian-Singh et al. also identified hypercoagulability as a risk factor, with thrombophilic disorders present in 28% of cases [34].

Brandt et al. and Boley et al. classify ischemic colitis into reversible, transient, chronic ulcerative, ischemic stricture, gangrene, and fulminant forms. Clinically, it is divided into gangrenous (15–20%) and non-gangrenous (80–85%) types. Chronic segmental colitis occurs in 20–25% of cases, while colonic stricture, seen in 10–15%, is more common in elderly patients and has an insidious onset [35,36,37].

Elderly patients have a poor prognosis. Anon et al. found that the absence of hematochezia, tachycardia, peritonism, anemia, hyponatremia, and colonic stenosis are indicators of an unfavorable prognosis [38].

Multiple studies have established a link between comorbidities and the severity of ischemic colitis, highlighting that hypertension, aortic pathology, and peripheral vascular disease significantly contribute to its occurrence [39,40,41].

Our patient, a 70-year-old, has a medical history of hypertension and congestive heart failure, which are key risk factors. Given his age, cardiac history, and presenting abdominal pain, abdominal ischemia was a highly likely diagnosis upon his emergency room admission.

Hypertension alters the morphology and function of blood vessels, damaging the vessel wall through shear stress and circumferential stretching. This leads to endothelial damage, neointima formation, activation of inflammatory pathways, and structural changes in vascular smooth muscle cells.

In elderly patients with atherosclerosis, increased blood pressure can lead to the mobilization of a thrombus or embolus from the atheromatous plaque. Congestive heart failure contributes to a reduced cardiac output, peripheral hypoperfusion, hypotension, and arrhythmias, all of which are significant risk factors for thromboembolic events. Atherosclerosis, the most common cause of peripheral vascular disease, results in blood vessel stenosis and decreased blood flow, impairing the delivery of oxygen and nutrients to tissues. Aortic pathology identified in imaging studies is a significant risk factor for thromboembolism, given the large size of the aneurysm (7.3 cm in diameter) and its extensive length (18 cm).

The aneurysm’s extensive length along the abdominal aorta, from below the renal arteries to the bifurcation, could lead to thromboembolic lesions in any of its vascular branches. In addition to ischemic colitis caused by thrombosis of the inferior mesenteric artery, other branches may also be affected, such as the testicular arteries, impairing blood supply to the testes, or the iliac arteries, potentially causing limb ischemia, spinal ischemia, or ischemic vascular lesions in the pelvic organs.

Di Martino et al. suggested that intraluminal thrombus offers a protective effect by reducing tensile stress on the aortic wall [14], while Boyd et al. argued the opposite, finding intraluminal thrombus deposits at the site of aneurysm rupture [40,41].

Kazi et al.’s study on the impact of intraluminal thrombus on the cellular and structural components of abdominal aortic aneurysms concluded that the thrombus accelerates aneurysmal degeneration by promoting inflammation and contributing to smooth muscle matrix degradation and lysis [41].

Hemodynamic disturbances in abdominal aortic aneurysms are linked to a prothrombotic environment, which disrupts nutrient and oxygen transport, as confirmed by Biasetti et al. [42,43].

In the non-aneurysmal segment, blood flow is laminar, but it becomes turbulent in the aneurysmal portion, increasing shear stress, activating platelets, and promoting platelet microparticle formation and thrombosis [43,44,45,46]. The soluble von Willebrand factor also plays a key role by binding to platelets via glycoprotein 1ba receptors, further increasing shear stress—an effect not typically seen in classic primary hemostasis without vascular damage [47,48].

Hourmand-Ollivier et al. found embolic heart sources in 21 patients with non-gangrenous segmental ischemic colitis [49]. Reiner et al. suggest that clinical and pathological manifestations after vascular obstruction vary based on the number and size of mesenteric vessel anastomoses [23].

Ischemic colitis is more common in the left colon, particularly the splenic flexure and sigmoid, due to poor blood flow and limited collateral circulation, making it more vulnerable to shock, hypotension, and dehydration.

Su et al. recommend stool cultures for *Salmonella*, *Shigella*, *Campylobacter*, and *E. coli O157:H7* when ischemic colitis is suspected [50], but in our case, emergency laparotomy was needed due to rapid deterioration.

CT scan can show a thin, non-enhancing colonic wall with lumen dilation, indicating total vascular occlusion. It can also detect thrombus, transmural damage, strictures, pneumatosis, and mesenteric venous gas, all linked to a poor prognosis [49].

Mesenteric angiography aids in distinguishing mesenteric ischemia from ischemic colitis, particularly in mild cases or right colon involvement [4].

The combination of aneurysmal thrombosis, ischemic colitis, and ulcerative colitis is rare but pathologically significant. We consider three hypotheses in the pathogenesis of this clinical case:

- Patient with ulcerative colitis → pro-thrombotic status → thrombosis in a pre-existing aneurysm → embolization → superimposed ischemic colitis.
- Elderly patient with thrombosed aortic aneurysm → mesenteric emboli → ischemic colitis → severe symptoms → accidental discovery of coexisting ulcerative colitis.
- Systemic autoimmune disease (e.g., lupus, vasculitis) → predisposition to all three entities.

Managing comorbid cases like aneurysmal thrombosis and ischemic colitis demands an integrated, coordinated approach, with careful risk assessment critical to guiding treatment decisions.

Comorbidities, such as aneurysmal thrombosis and ischemic colitis, can worsen the primary disease’s course, making a multidisciplinary approach essential, with close collaboration among internal medicine, vascular and general surgery, radiology, gastroenterology, and emergency medicine specialists.

Management of these patients involves both prophylactic and therapeutic strategies targeting thromboembolic risk and intestinal ischemia. Optimizing anticoagulation, tailored to renal function and hemodynamic status, is essential, while specific anti-inflammatory treatment for ischemic colitis may be needed, potentially complicating anticoagulation management. Close monitoring for complications is crucial, as medications can increase the risk of adverse events like bleeding. Early assessment and intervention, along with patient education on warning signs and lifestyle adjustments, are key to optimizing treatment outcomes. When conservative treatments fail to improve the patient’s condition, surgery becomes a viable option. Indications for surgery include intestinal necrosis or ischemic colitis unresponsive to medical therapy. Surgical procedures may include segmental intestinal resections to remove affected areas, and in severe cases, a colostomy may be necessary. Effective management of ischemic colitis extends beyond immediate intervention, requiring a tailored long-term plan to improve quality of life and prevent serious complications (Table 3, Table 4 and Table 5).

This case study offers valuable insights into the pathology of ischemic colitis and enhances the understanding of available treatment options. The points discussed align with the paper’s central theme, highlighting the link between aneurysmal thrombosis and ischemic colitis, and advocating for an integrated approach to their diagnosis and treatment. This case of ischemic colitis associated with a thrombosed abdominal aortic aneurysm provides key clinical insights. Although ischemic colitis is typically linked to low-flow states or small vessel disease, this report underscores the rare yet significant vascular cause of large-vessel thrombosis leading to mesenteric hypoperfusion.

## 4. Conclusions

This case highlights ischemic colitis as a vascular disorder, highlighting the need for a broader differential diagnosis when common causes are unclear. Timely imaging, a multidisciplinary approach, and attention to vascular risks are key to identifying rare causes like aneurysmal thrombosis. While thrombosed abdominal aortic aneurysms can cause mesenteric ischemia, their link to ischemic and ulcerative colitis is unique, emphasizing the importance of accurate risk assessment in treatment planning.

## Figures and Tables

**Table 1:** Laboratory tests upon admission and after surgery.

Laboratory Tests	Laboratory Tests upon Admission	Laboratory Tests After Surgery
White blood cell	17.20 × 10^3^ cells/μL	28 × 10^3^ cells/μL
Neutrophil proportion	89.4%	94%
Hemoglobin	14.2 g/dL	12.8 g/dL
Platelet count	147 × 10^3^ cells/μL	125 × 10^3^ cells/μL
Creatinine	1.9 mg/dL	2.5 mg/dL
Urea	107 mg/dL	180 mg/dL
INR	1.34	1.15
Prothrombin time	14.7 s	14 s
Fibrinogen	1335 mg/dL	1500 mg/dL
Erythrocyte sedimentation rate	55/mm	50/mm

**Table 2:** Arterial blood gas analysis.

Parameter	Upon Admission to the Intensive Care Unit	6 h After Admission	10 h After Admission
pH	7.38	7.25	7.20
PaCO_2_	25 mmHg	60 mmHg	38 mmHg
HCO_3_⁻	15 mEq/L	18 mEq/L	14 mEq/L
PaO_2_	95 mmHg	70 mmHg	75 mmHg
SaO_2_	98%	92%	94%
BE	−8 mmol/L	−6 mmol/L	−15 mmol/L
Lactat	1.5 mmol/L	2.0 mmol/L	3 mmol/L
Comments	Totally compensated respiratory metabolic acidosis	Ph is predominantly acidified by respiration	Severe pure metabolic acidosis

Legend: PaCO_2_ = partial pressure of carbon dioxide, HCO_3_⁻ = bicarbonate ion, PaO_2_ = alveolar partial pressure of oxygen, BE = basic excess.

**Table 3:** Prevention and treatment strategies.

1. Address Risk Factors
• Cardiovascular risk management: ○ Control hypertension, hyperlipidemia, and diabetes. • Consider antiplatelet agents (e.g., aspirin) for vascular disease history, but use cautiously due to potential GI side effects. • Quit smoking to enhance vascular health. • Ensure hydration, especially in elderly or hospitalized patients, to maintain proper perfusion.
2. Medication Assessment
• Avoid or carefully monitor: Vasoconstrictors (e.g., decongestants, ergotamine); NSAIDs, digoxin, diuretics, and constipation-inducing drugs. ○ These can reduce colonic blood flow or contribute to ischemia.
3. Surgical Prevention (in Rare Cases)
Patients with known mesenteric arterial disease might benefit from angioplasty/stenting or bypass surgery to improve blood flow.
4. Management of Mild Ischemic Colitis
Supportive care: ▪ Bowel rest, IV fluids, and broad-spectrum antibiotics; ▪ Avoid laxatives or agents that reduce bowel motility. Most mild/moderate cases resolve without surgery.
5. Management of Severe Cases
• Surgery (e.g., partial colectomy) may be required if there is one of the following: ○ Perforation, necrosis, or persistent bleeding.

Legend: NSAIDs = nonsteroidal anti-inflammatory drug, CRP = C-reactive protein, ESR = erythrocyte sedimentation rate, CBC = complete blood count, MRA = magnetic resonance angiography, CTA = computed tomography angiography, GI = gastrointestinal.

**Table 4:** Early diagnosis tools.

1. Clinical Suspicion
• Early recognition of symptoms: ○ Sudden onset abdominal pain (typically left-sided) ○ Bloody diarrhea or rectal bleeding ○ Tenderness without signs of peritonitis (in early/mild cases) • Often follows episodes of hypotension, dehydration, or recent surgery
2. Laboratory Tests
CBC: May show leukocytosis or anemia (if bleeding is present) Lactat: Elevated in severe ischemia CRP/ESR: Elevated inflammatory markers When lab results are not specific—adjunctive tools are critical
3. Imaging
• CT abdomen with contrast (most useful non-invasive tool): ○ Shows colonic wall thickening, thumbprinting (submucosal edema/hemorrhage), pneumatosis, or portal venous gas in severe cases
4. Colonoscopy or Flexible Sigmoidoscopy
• Can provide direct visualization and biopsy: ○ Pale mucosa, friability, ulceration • Risk of perforation in severe disease, so used cautiously • Allows for definitive diagnosis if performed early on and safely
5. Doppler Ultrasound/MRA/CTA
For assessing mesenteric blood flow in patients with recurrent symptoms or suspected chronic ischemia

Legend: NSAIDs = nonsteroidal anti-inflammatory drug, CRP = C-reactive protein, ESR = erythrocyte sedimentation rate, CBC = complete blood count, MRA = magnetic resonance angiography, CTA = computed tomography angiography, GI = gastrointestinal.

**Table 5:** Clinical takeaways.

Approach	Examples
Preventive Treatment	Cardiovascular control, medication adjustment, hydration
Medical Management	IV fluids, bowel rest, antibiotics
Surgical Intervention	For complications or mesenteric vascular disease
Clinical Monitoring	Recognize pain, bleeding, risk factors
Imaging	CT of abdomen with contrast (first line)
Endoscopy	Visual and biopsy confirmation (if stable)
Vascular Studies	CTA, MRA, Doppler for chronic/recurrent or high-risk patients

Legend: MRA = magnetic resonance angiography, CTA = computed tomography angiography, IV = intravenous.

## Data Availability

The data presented in this study are available on request from the corresponding author. The data are not publicly available due to patient confidentiality.
